# Remarkable Case of Palsy

**Published:** 1816-11

**Authors:** Peter Miller

**Affiliations:** Surgeon.


*For the London Medical and Physical Journal.*

### *Remarkable Case of Palsy;* by Mr. PETER MILLER, Surgeon.

THE case of Palsy given by Dr. Cross, of Glasgow, in your last Number, brought to my recollection the following case.

George Reid, weaver, Main-street, Gorbals, twenty-nine years of age, married, red hair, sanguine temperament, long neck, head of ordinary size, 5 feet 6¼ inches high, served as a private in the 27th regt. eleven years in Sicily and in the Peninsula, until he was wounded in the right arm at the heights of Ardeal. During this period he had fever thrice, had now and then indulged pretty freely in the glass, and is always sooner inebriated than his companions. 20th July last, after eating a supper of bread, ham, and tea, before going going to bed, he had occasion to go down stairs, when, in the middle of the first flight, a cramp pain ascended from the right ham upwards to the back of the neck, which made him wheel round, and fall on his back. He immediately uttered screams, which brought his friends to his assistance. He was found in a state of insensibility—his face *blue*, and his breathing could scarcely be heard. In about a quarter of an hour after the attack, I saw him. He was still insensible, and was supported on his left leg by his friends. In this state he was carried up-stairs, with his right leg trailing after him. The pupils were dilated, and he had a wild staring look; his extremities were cold; pulse 75, very full: in short he had complete hemiplegia.

While his friends held him on a chair, I opened a vein in the right arm, and, in a full stream, drew ℥xxxviii of blood: towards the end he became faintish, and cold water being dashed in his face, he, for the first time, became a little sensible. This being over, he had recovered the use of his right side so much as to require but little assistance in going to his bed. A dose of x gr. calomel and ʒſs jalap was given.

On the 21st, the physic had operated well. He had slept well, and the right side had lost nothing since the following night, although it was by no means equal in strength to the left. ℥xx of blood were drawn from the left arm in a large stream. He stood this well, and, immediately after, the freedom of *motion* of the right limbs was equal to the left, although he felt the whole very weak. In about an hour, without my knowledge at the time, he rose and put on his clothes, went out, and walked about a quarter of a mile, as he said, "without the smallest halt." In the evening the purgative was repeated, and operated well.

On the 22d he resumed the loom, and gradually recovered his former strength—has continued healthy ever since.

47, *Adelphi-street, Glasgow;*
*23d Sept.* 1816.

